# Theranostic Value of Multimers: Lessons Learned from Trimerization of Neurotensin Receptor Ligands and Other Targeting Vectors

**DOI:** 10.3390/ph10010029

**Published:** 2017-03-10

**Authors:** Simone Maschauer, Jürgen Einsiedel, Dominik Reich, Harald Hübner, Peter Gmeiner, Hans-Jürgen Wester, Olaf Prante, Johannes Notni

**Affiliations:** 1 Department of Nuclear Medicine, Molecular Imaging and Radiochemistry, Friedrich-Alexander University Erlangen-Nürnberg (FAU), Schwabachanlage 6, 91054 Erlangen, Germany; olaf.prante@uk-erlangen.de; 2 Department of Chemistry and Pharmacy, Medicinal Chemistry, Emil Fischer Center, Friedrich-Alexander University Erlangen-Nürnberg (FAU), Schuhstraße 19, 91052 Erlangen, Germany; jürgen.einsiedel@fau.de (J.E.); harald.hübner@fau.de (H.H.); peter.gmeiner@fau.de (P.G.); 3 Lehrstuhl für Pharmazeutische Radiochemie, Technische Universität München, Walther-Meißner-Strasse 3, 85748 Garching, Germany; dominik.reich@tum.de (D.R.); h.j.wester@tum (H.-J.W.); johannes.notni@tum.de (J.N.)

**Keywords:** neurotensin, neurotensin receptor, NTS1, positron emission tomography, PET, gallium-68, TRAP, multimerization

## Abstract

Neurotensin receptor 1 (NTS1) is overexpressed on a variety of cancer entities; for example, prostate cancer, ductal pancreatic adenocarcinoma, and breast cancer. Therefore, it represents an interesting target for the diagnosis of these cancers types by positron emission tomography (PET). The metabolically-stabilized neurotensin (NT) derivative peptide Nlys^8^-Lys^9^-Pro^10^-Tyr^11^-Tle^12^-Leu^13^-OH was elongated at the N-terminus with 6-azido norleucine and coupled with the 1,4,7-triazacyclononane-1,4,7-tris[(2-carboxyethyl)methylenephosphinic acid] (TRAP) chelator TRAP(alkyne)_3_ in order to synthesize a NT trimer with subnanomolar affinity and high stability. The ^68^Ga-labeled peptide [^68^Ga]Ga-TRAP(NT4)_3_ was characterized in vitro using the NTS1-expressing human colorectal adenocarcinoma cell line HT29. It displayed fast and high internalization rates of >90%, but also fast efflux rates of 50% over 15 min. In vivo, [^68^Ga]Ga-TRAP(NT4)_3_ showed moderate HT29 tumor uptake values of 1.7 %ID/g at 60 min post-injection (p.i.), but also high uptake and retention in the kidneys and liver. A comparison of data for trimer/monomer pairs of NT ligands and other targeting vectors (peptides and peptoids targeting integrins α_v_β_3_, α_5_β_1_, and α_v_β_6_, the PSMA-ligand DUPA (2-[3-(1,3-dicarboxypropyl)-ureido]pentanedioic acid), and nitroimidazoles targeting hypoxia) revealed that multimers always exhibit higher target affinities and tumor uptake, but not necessarily improved tumor-to-tissue ratios. Thus, although in vitro data are not suitable for prediction of in vivo performance, multimers are potentially superior to monomers, particularly for applications where high tumor accumulation is crucial.

## 1. Introduction

The neurotensin (NT) receptor, especially neurotensin receptor 1 (NTS1) is described to be overexpressed in a variety of cancer entities [1], e.g., pancreatic ductal adenocarcinoma [2], non-small cell lung cancer [3], breast cancer [4], and prostate cancer [5], whereas it shows only low expression in the tissue from which these tumors arise. Neurotensin, a peptide consisting of 13 amino acids, binds with high affinity to this G-protein-coupled receptor. Therefore, it was considered as a possible molecular agent for (radio)therapy and/or diagnosis of cancer by targeting NTS1 [6,7]. The shortest binding sequence of NT to NTS1 is the C-terminal fragment NT(8–13) with the amino acids Arg^8^-Arg^9^-Pro^10^-Tyr^11^-Ile^12^-Leu^13^-OH. This peptide sequence is rapidly degraded in vivo with a biological half-life of only a few minutes, or even less [8,9,10]. Consequently, great efforts were taken regarding stabilization of this peptide sequence. It was found that the cleavage sites are located between Arg^8^-Arg^9^, Pro^10^-Tyr^11^ and Tyr^11^-Ile^12^. Hence, NT analogs developed for in vivo application were mainly modified at these amino acids and stabilization was attempted by modifying the backbone of the peptide [11,12,13]. Most NT analogs published until now were labeled with the SPECT (single-photon emission computer tomography) radionuclides technetium-99m or indium-111, whereas only a few studies still focused on the development of NT analogs for imaging using positron emission tomography (PET) [6,14], an imaging modality with higher sensitivity compared to SPECT [15]. The positron emitter gallium-68 is highly suitable and frequently used for the convenient labeling of chelator-linked peptides, since it is highly available in institutions without a cyclotron, due to its production through germanium-68 (half-life of 271 days) in a generator system. Among the NT radiopeptides for PET imaging, only the peptides with the amino acid sequence Nlys^8^-Lys^9^-Pro^10^-Tyr^11^-Tle^12^-Leu^13^-OH developed by our group were reported to have high stability not only in vitro, but also in vivo [16,17,18].

Two approaches to enhance stability were undertaken by multimerization leading to dimers or tetramers with unmodified NT(8–13) sequences [19,20]. The NT(8–13) tetramer comprising cyclam as a chelator and labeled with copper-64, had a biological half-life of 34 min in rat plasma and 60 min in mouse plasma. Furthermore, it displayed high tumor uptake in an HT29 tumor mouse model, but also low blood clearance, probably due to the formation of a metabolite, which was postulated to be the ^64^Cu-cyclam-tetraarginine complex [19]. A relatively new approach for multimerization of biomolecules is the use of 1,4,7-triazacyclononane-1,4,7-tris[(2-carboxyethyl)methylenephosphinic acid] (TRAP) ligands [21,22], which not only simplify multimerization, but are also excellent chelators for labeling with gallium-68 [23,24,25]. Compared to other chelators, only very low amounts of the TRAP ligands are necessary for ^68^Ga-labeling in high radiochemical yields and, therefore, high molar radioactivities of >1000 GBq/µmol are achievable.

Consequently, it was our intention here to combine the TRAP methodology with our metabolically-stabilized NT(8–13) sequence Nlys^8^-Lys^9^-Pro^10^-Tyr^11^-Tle^12^-Leu^13^-OH (NT4) to gain a trimeric NT ligand with outstanding affinity and stability. The synthesis, radiosynthesis, as well as in vitro characterization using HT29 cells of the new peptide [^68^Ga]Ga-TRAP(NT4)_3_, are described. Furthermore, [^68^Ga]Ga-TRAP(NT4)_3_ was evaluated in vivo using HT29 xenografted nude mice in biodistribution and small animal PET studies to study its applicability as an imaging agent of NTS1-positive tumors for PET.

## 2. Results

### 2.1. Syntheses and Radiosyntheses

**Figure 1:**
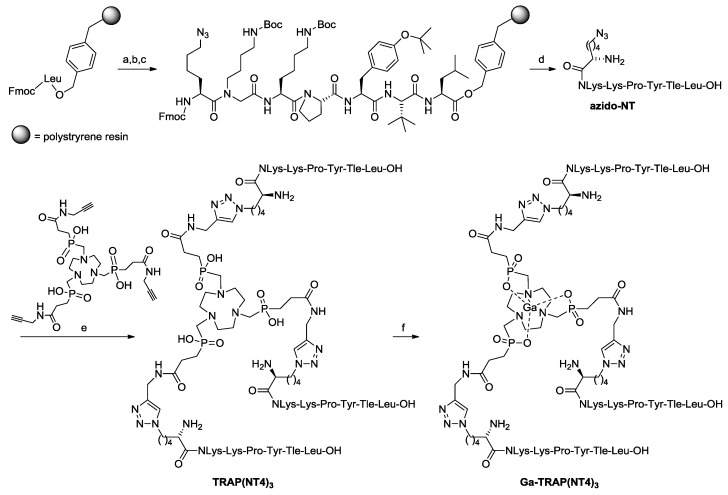
Synthesis of Ga-TRAP(NT4)_3_, reagents and conditions: (**a**) Fmoc-deprotection: piperidine/DMF (1:4), μ∼: 5× 5 s, 100 W, 5× cooling to −10 °C; (**b**) peptide coupling for Fmoc-Tle-OH, Fmoc-Tyr(tBu)-OH, Fmoc-Pro-OH, Fmoc-Lys(Boc)-OH: Fmoc-AA-OH, PyBOP, DIPEA, HOBt, DMF, μ∼, 15× 10 s, 50 W, 15× cooling to −10 °C; (**c**) for *N*-Fmoc-*N*-(4-Boc-aminobutyl)-Gly-OH, Fmoc-Nle(6-N_3_)-OH: Fmoc-AA-OH, HATU, DIPEA, DMF, μ∼-assisted coupling (see **b**); (**d**) deprotection/cleavage of the peptide: TFA/anisole/phenol 83:12:5, reaction time (r.t.), 3 h, followed by RP-HPLC; (**e**) (1) Cu(OAc)_2_, sodium ascorbate, H_2_O/MeOH, r.t., 1 h; (2) NOTA, HCl aq., pH 3, r.t., 10 days, followed by RP-HPLC; and (**f**) Ga(NO_3_)_3_, H_2_O, r.t., 5 min or [^68^Ga]GaCl_3_, HEPES, pH 2.5–3, 98 °C, 5 min.

The synthesis of the azide functionalized peptide was based on our previous publications on metabolically-stabilized NT(8–13) derivatives (Figure 1) [16,17,26,27]. Starting from Fmoc-leucinyl Wang resin, we applied solid-phase methods with repetitive cycles of Fmoc deprotection using piperidine and acylation with the respective Fmoc-protected amino acids. Amino acid activation was done in the presence of PyBOP/HOBt, allowing the incorporation of *tert*-leucine, tyrosine, proline, and lysine. The more powerful coupling agent HATU was employed for the attachment of *N*-(4-aminobutyl)-glycine and for 6-azido-norleucine. Microwave acceleration proved to be advantageous for both Fmoc deprotection and acylation reactions. Cleavage from the resin with TFA in the presence of anisole and phenol resulted in the formation of crude H-Nle(6-N_3_)-NLys-Lys-Pro-Tyr-Tle-Leu-OH, which was purified by preparative high-performance liquid chromatography (HPLC).

Conjugation of the azide-functionalized neurotensin derivative azido-NT (H-Nle(6-N_3_)-NLys-Lys-Pro-Tyr-Tle-Leu-OH) to a three-fold alkyne-functionalized derivative of the TRAP [25,28,29] chelator, TRAP(alkyne)_3_ [21], was conducted conveniently by copper-catalyzed azide-alkyne cycloaddition (CuAAC), followed by the removal of Cu^II^ from the conjugate by means of transchelation (Figure 1) [30].

The radiosynthesis of [^68^Ga]Ga-TRAP(NT4)_3_ was performed following our previously published protocol [25]. Only 1 nmol (0.8 µM) of the labeling precursor TRAP(NT4)_3_ was necessary to achieve high radiochemical yields of >98% after 5 min at 98 °C in HEPES buffer at pH 2.5, resulting in molar radioactivities of 80–120 GBq/µmol at the end of synthesis (EOS). For in vitro and in vivo experiments the radiotracer was purified by SPE and diluted with saline.

### 2.2. In Vitro Evaluation

The receptor affinity of Ga-TRAP(NT4)_3_ to NTS1 and NTS2 was determined by a competitive binding assay using [^3^H]neurotensin and [^3^H]NT(8–13) as radioligands and cell membrane homogenates containing the respective human receptors [31]. TRAP(NT4)_3_ and the stabilized monomeric peptide Pra-NLys-Lys-Pro-Tyr-Tle-Leu-OH (NT4) were used for comparison [17]. The results are listed in Table 1. Ga-(TRAP)_3_ showed subnanomolar affinities of 0.12 nM and 0.21 nM to NTS1 and NTS2, respectively, and the affinity for the labeling precursor TRAP(NT4)_3_ was in the same range with a minor selectivity for NTS2 over NTS1. Compared to the monomer NT4 [17], the affinity for the trimers is, as expected, significantly higher (factors of 10–50), however, for the Ga-complex a similar binding behavior for NTS1 and NTS2 could be observed (Table 1).

**Figure 2:**
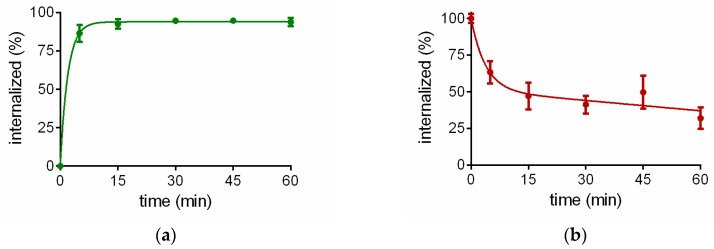
(**a**) Internalization rate of [^68^Ga]Ga-TRAP(NT4)_3_ in HT29 cells in vitro; (**b**) efflux of [^68^Ga]Ga-TRAP(NT4)_3_ from HT29 cells after internalization for 30 min. Each data point represents the mean ± standard deviation of three experiments performed in quadruplicate.

^68^Ga-TRAP(NT4)_3_ showed a fast internalization in NTS1-expressing HT29 cells. After 5 min the maximum internalization rate of 87% was already achieved and stayed constant over the time period of 60 min (Figure 2a). After washing the cells to remove non-internalized radioligand, fresh medium was added in order to determine the efflux rate. As shown in Figure 2b, efflux was fast at the beginning, with 50% efflux after only 15 min.

### 2.3. In Vivo Evaluation of [^68^Ga]Ga-TRAP(NT4)_3_

**Figure 3:**
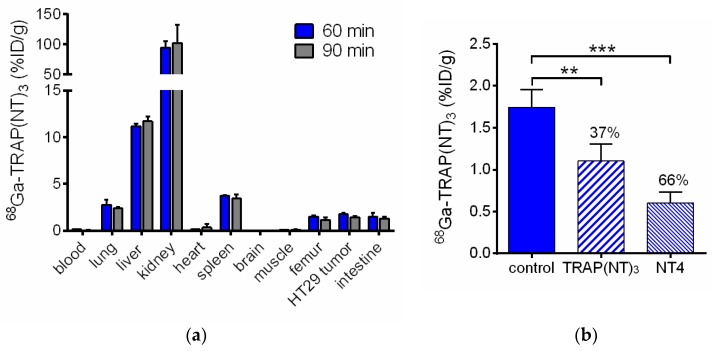
(**a**) Biodistribution of [^68^Ga]Ga-TRAP(NT4)_3_ in HT29 tumor-bearing mice at 60 and 90 min p.i. (*n* = 3); and (**b**) blocking of the HT29 tumor by co-injection with TRAP(NT4)_3_ (20 nmol, (*n* = 3)) or NT4 (100 nmol; (*n* = 2)) at 60 min p.i. in comparison with control animals. Each bar represents the mean ± standard deviation. ** *p* = 0.0022, *** *p* = 0.0004 (unpaired *t*-test).

For in vivo evaluation of [^68^Ga]Ga-TRAP(NT4)_3_ the NTS1-positive human colorectal adenocarcinoma cells HT29 were injected in nude mice to generate a subcutaneous tumor. The radioligand was injected in the tail vein of the mice and the biodistribution was determined 60 and 90 min after tracer injection. The results are given in Table 2 and Figure 3. While only negligible radioactivity was measured in the blood, the highest uptake values of 55–102 %ID/g were observed in the kidneys and moderate uptake was observed in the liver with values of 11–12 %ID/g at 60 and 90 min post-injection (p.i.). The clearance rate of [^68^Ga]Ga-TRAP(NT4)_3_ in these excretion organs was apparently low. Actually, almost no washout or clearance could be seen in any organ besides blood, leading to increasing tumor-to-blood ratios of 13 at 60 min p.i. to 36 at 90 min p.i. The uptake in the tumor was 1.74 %ID/g after 60 min and showed good retention until 90 min p.i. (1.44 %ID/g). Blocking experiments were performed to confirm the specificity of the tumor uptake of [^68^Ga]Ga-TRAP(NT4)_3_ in vivo. Therefore, TRAP(NT4)_3_ (20 nmol) or NT4 (100 nmol) were co-injected together with the radiotracer to define the nonspecific uptake of the tracer in the tumor region. The tumor uptake under these blocking conditions was significantly reduced by 37% with TRAP(NT4)_3_ and by 66% with NT4 (Figure 3b). Apart from the intestine, the uptake in all other organs was not affected by the blocking substances (Table 2), confirming the receptor-mediated uptake of [^68^Ga]Ga-TRAP(NT4)_3_, as it was reported that NTS receptors are expressed in the small intestine of mice [32].

The in vivo stability of [^68^Ga]Ga-TRAP(NT4)_3_ was measured from a blood sample taken 5 min after injection in mice. HPLC analysis showed the formation of a more hydrophilic radiometabolite by 8% (data not shown). Unfortunately, it was not possible to determine the stability at later time points due to the low amount of residual radioactivity in the blood.

**Figure 4:**
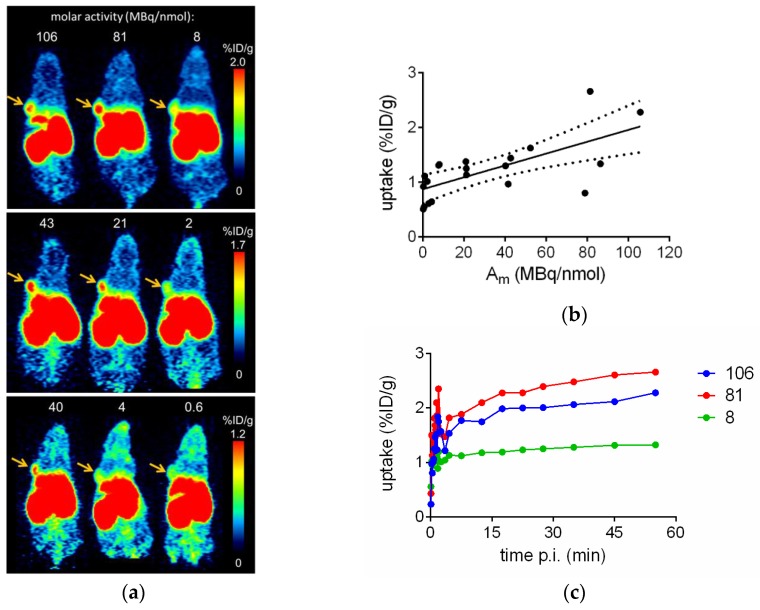
(**a**) Small animal PET scans of three different HT29-bearing nude mice each injected with varying amounts of [^68^Ga]Ga-TRAP(NT4)_3_ on three consecutive days, resulting in different molar radioactivities at the time of injection. Depicted are frames from 40 to 60 min; arrows indicate the tumors; (**b**) HT29 tumor uptake at 50–60 min p.i. expressed as percent of injected dose per g (%ID/g) of [^68^Ga]Ga-TRAP(NT4)_3_ dependent of molar radioactivity (A_m_) of eight mice each scanned two to three times. Given are single data values with the 95% confidence bands for the best fit line; and (**c**) time activity curves showing uptake of [^68^Ga]Ga-TRAP(NT4)_3_ in the HT29 tumor dependent of molar radioactivity in MBq/nmol.

Figure 4a shows representative small animal PET scans of three different HT29 tumor-bearing mice injected with [^68^Ga]Ga-TRAP(NT4)_3_ at 40–60 min p.i. The highest tumor uptake values of approximately 2 %ID/g were determined with molar radioactivities of more than 20 MBq/nmol. The respective time activity curves (TACs) showed fast uptake in the tumor which steadily increases over time from 5 to 60 min p.i. when using high molar radioactivities of 81 or 106 MBq/nmol, or remained constant when using low molar radioactivities of 8 MBq/nmol (Figure 4c). Considering all eight mice, which were repeatedly measured by PET with varying molar radioactivities of [^68^Ga]Ga-TRAP(NT4)_3_, a good correlation of molar radioactivity and tumor uptake could be observed (Figure 4b; *r* = 0.713, Spearman correlation, *p* < 0.0004).

## 3. Discussion

### 3.1. Characteristics of the Neurotensin Ligand Trimer [^68^Ga]Ga-TRAP(NT4)_3_

The trimeric peptide [^68^Ga]Ga-TRAP(NT4)3 showed receptor affinities in the subnanomolar range with values of 0.1 and 0.2 nM for NTS1 and NTS2, respectively. In addition, the in vitro characteristics of [^68^Ga]Ga-TRAP(NT4)_3_ determined in HT29 cell binding assays, especially the high and fast internalization rate of almost 90% after only 5 min, which stayed constant over at least 60 min, and the moderate efflux rate, were highly promising that [^68^Ga]Ga-TRAP(NT4)_3_ could be a PET ligand for imaging of neurotensin receptors in vivo. Therefore, we studied [^68^Ga]Ga-TRAP(NT4)_3_ in our established HT29 tumor-bearing nude mice model.

Our previously published monomers comprising the same amino acid sequence as the trimeric [^68^Ga]Ga-TRAP(NT4)_3_ showed standard uptake values (SUVs) in HT29 tumors between 0.14 and 0.36 at 60 min p.i. [18]. Compared with these, [^68^Ga]Ga-TRAP(NT4)_3_ showed a significantly higher tumor uptake of 1.7 %ID/g, which equals an SUV of about 0.7. The clearance of [^68^Ga]Ga-TRAP(NT4)_3_ from blood was highly similar to that of monomers (monomers: 0.05–0.26 %ID/g [18], [^68^Ga]Ga-TRAP(NT4)_3_: 0.13 %ID/g). Together with the increased tumor uptake of the trimer compared to monomeric tracers, the resulting tumor-to-blood ratios were high and increasing over time, allowing excellent visualization of the tumors by small animal PET. The limitation of [^68^Ga]Ga-TRAP(NT4)_3_ lays mainly in the extremely high uptake in the kidneys, and also the high uptake in the liver, spleen, and lungs, with no or only minute washout or clearance from these organs.

### 3.2. Putting Trimerization into Perspective: Comparison of Various Ligand Monomers and Their TRAP Trimers

Essentially, our experimental results show that trimerization of NT ligands resulted in marked improvement of in vitro properties, which, however, did not translate into better in vivo performance, such as improved tumor-to-organ ratios. In order to rate this outcome, it is quite helpful to compare the available data for other structurally-related systems.

Table 3 summarizes in vitro and in vivo data for Ga-labeled symmetrical TRAP trimers and structurally-related monomers, that is, 1:1 conjugates with other gallium chelators. Data were taken from the literature and complemented with unpublished data if applicable. The close structural relationship between these monomer-trimer pairs and the similarity of experimental conditions (namely, tumor models used for evaluation) allows drawing conclusions on the general utility of multimerization, widely independent from scaffold-related effects.

Most obviously, all of the trimeric compounds given in Table 3 showed improved affinities to their targets in comparison with the respective monomers. However, the range of these improvements varied considerably between a five-fold and 435-fold enhancement compared to the monomeric compounds. This could be due to differences in the density of homodimer receptors in the cells of the in vitro assay that has been used. The direct proximity of the neighboring receptor is a prerequisite for the high affinity of a trimer, since rebinding of the radioligand is most likely. The rebinding mode of trimeric ligands, being distinct from the bivalent-binding mode, as it is known from heterodimer-selective GPCR ligands [33], could be one of the most important factors that improves the tumor retention of trimeric radioligands in vivo. Indeed, for all trimers under consideration, this translated to improved tumor uptakes compared to the monomers, while a quantitative correlation between enhancement of affinity and tumor accumulation is not found.

**Figure 5:**
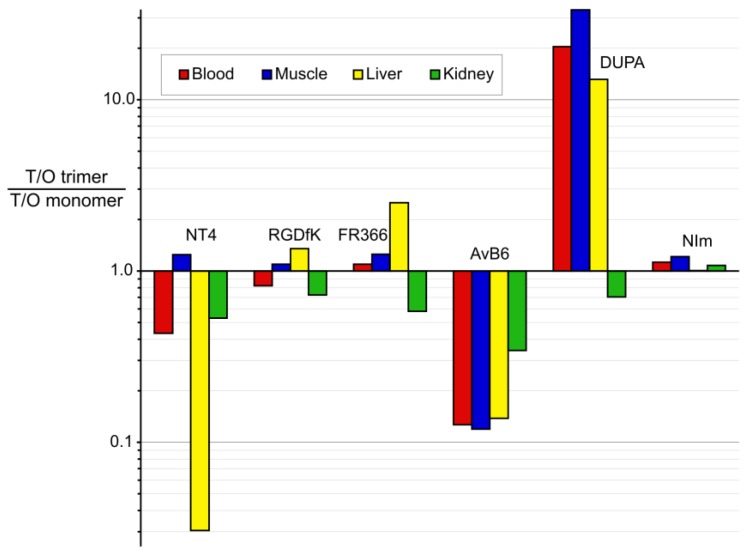
Quotients of tumor-to-organ ratios (T/O) of trimers and corresponding monomers, respectively, for compounds described in Table 3. Values above 1 indicate improvement upon trimerization, while values below 1 translate to deterioration.

However, Figure 5 shows that the impact of multimerization on overall in vivo performance is another story. There is no systematic pattern for improvement or deterioration of tumor-to-organ/tissue ratios upon trimerization, apart from the finding that it entails a somewhat less favorable tumor-to-kidney ratio in most cases. This is most probably related to slower renal excretion of the considerably larger trimers. For the nitroimidazole derivatives, where differences in molar weights are much less pronounced, tumor/kidney ratios are expectedly almost the same.

The different impact of trimerization on other T/O ratios is puzzling, and dramatic changes can be observed in either direction. Generally, compound polarity (expressed as log*D*_7.4_) can apparently not be made responsible for these observations, because for most monomer/trimer pairs, the values are quite similar. There is one exception, AvB6; here, the trimer is considerably less hydrophilic compared to the monomer, which increases the unspecific retention and apparently shifting excretion to the hepatobiliary pathway [34]. On the other hand, the dramatically improved in vivo performance of the DUPA trimer was found earlier to be rooted in the metabolic instability of the DUPA structure [35]. In analogy to observations made for BBN(7–14) dimers [36], the presence of three copies of the unstable targeting vector DUPA apparently helps to maintain the functionality (i.e., targeting properties) of the entire construct over a longer time and, thus, enhances apparent in vivo stability [21].

Overall, it can be concluded that the outcome of multimerization is hardly predictable. Despite being associated with higher target affinity and tumor uptake, the in vivo behavior of multimers is apparently governed by other factors to an even larger extent. Basic in vitro data, such as receptor affinities, thus appears to be unsuitable for prediction of overall performance. Hence, it is not entirely surprising that the performance of the NT ligand trimer [^68^Ga]Ga-TRAP(NT4)_3_ is below expectations, despite excellent in vitro data. Only the dramatically enhanced liver uptake appears to be related to a yet unknown but specific mechanism, which deserves more detailed investigation in the future.

## 4. Materials and Methods

### 4.1. General

9-Fluorenylmethoxycarbonyl (Fmoc) protected amino acids and Fmoc-leucinyl-Wang resin were purchased from Novabiochem of the Merck Millipore Group (Darmstadt, Germany) and from Bachem AG (Bubendorf, Switzerland). *N*-Fmoc-*N*-(4-Boc-aminobutyl)-Gly-OH (Fmoc-NLys(Boc)-OH) was purchased from Aldrich (Taufkirchen, Germany) and Fmoc-6-azido-norleucine (Fmoc-6-azido-Nle) from Iris Biotech (Marktredwitz, Germany). All other organic reagents were obtained from VWR International GmbH (Darmstadt, Germany) or Sigma-Aldrich Chemie GmbH (Munich, Germany). The solid phase peptide synthesis (SPPS) was conducted using a microwave assisted protocol (Discover microwave reactor, CEM GmbH, Kamp-Lintfort, Germany) starting from Fmoc-Leu-Wang resin. The reactions were carried out in a silanized glass tube loosely sealed with a silicon septum. Remark: the development of overpressure was avoided by using DMF as the solvent and intermittent cooling in an ethanol-ice bath. The following amino acids were incorporated as their commercially available derivatives: *N*-α-Fmoc-*tert*-leucine (Fmoc-Tle-OH), *N*-α-Fmoc-*O*-*tert*-butyl-tyrosine (Fmoc-Tyr(tBu)-OH), Fmoc-proline (Fmoc-Pro-OH), *N*-α-Fmoc-*N*-ε-Boc-lysine (Fmoc-Lys(Boc)-OH), *N*-Fmoc-*N*-(4-Boc-aminobutyl)-Gly-OH (Fmoc-NLys(Boc)-OH) and *N*-α-Fmoc-6-azidonorleucine (Fmoc-6-azido-Nle-OH). Elongation of the peptide chain was done by repetitive cycles of Fmoc deprotection and subsequent couplings of the respective amino acids. Fmoc deprotection was performed by treating the resin with 25% piperidine in DMF (microwave irradiation: 7 × 5 s, 100 W), followed by washings with DMF (5×). The building blocks and reagents (as specified below) were dissolved in a minimum amount of DMF and the irradiation was performed 15 × 10 s employing 50 W. In between each irradiation step, cooling of the reaction mixture to a temperature of −10 °C was achieved by sufficient agitation in an ethanol–ice bath. After the last acylation step and Fmoc deprotection, the resin was 10× rinsed with CH_2_Cl_2_ and dried in vacuo. The cleavage from the resin was done using a mixture of trifluoroacetic acid (TFA)/anisole/phenol 83:12:5 for 3 h. After evaporation of the solvent and precipitation in *tert*-butylmethylether, the crude peptide was purified using preparative RP-HPLC (Agilent 1100 preparative series (Agilent Technologies, Waldbronn, Germany), column: Zorbax Eclipse XDB-C8, 21.2 mm × 150 mm, 5 μm particles, flow rate 10 mL/min) applying a linear gradient. After the separation, the peptide (formate salt) was lyophilized and peptide purity and identity were assessed by analytical HPLC (Agilent 1100 analytical series, equipped with a quaternary pump and variable wavelength detector detector; column: Zorbax Eclipse XDB-C8 analytical column, 4.6 mm × 150 mm, 5 μm, flow rate: 0.5 mL/min) coupled to a Bruker Esquire 2000 mass detector equipped with an ESI-trap (Bruker Daltonics, Bremen, Germany).

### 4.2. Synthesis of H-Nle(6-N_3_)-NLys-Lys-Pro-Tyr-Tle-Leu-OH (azido-NT)

The amino acids were incorporated successively using the coupling conditions AA/PyBOP/diisopropylethylamine (DIPEA)/1 hydroxybenzotriazole (HOBt) (5 eq (equivalent)/5 eq/5 eq/7.5 eq) for Fmoc-Tle-OH, Fmoc-Tyr(tBu)-OH), Fmoc-Pro-OH and 2× Fmoc-Lys(Boc)-OH. The coupling conditions AA/HATU/DIPEA were used for Fmoc-NLys(Boc)-OH (4 eq/4 eq/4 eq) and Fmoc-ε-azido-Lys (5 eq/5 eq/5 eq). Further treatment of the resin was done as described in “General”. Preparative RP-HPLC gradient: CH_3_CN in H_2_O (0.1% HCO_2_H) 10%–25% in 9 min (*t*_R_: 7.5 min). Analytical HPLC: linear gradient 10%–40% CH_3_OH in H_2_O + 0.1% HCO_2_H in 18 min, purity: >99% (*t*_R_: 16.3 min). ESI-MS: [M + H]^+^; calcd for C_44_H_75_N_12_O_9_: 916.1, found: 915.7.

### 4.3. Synthesis of TRAP(NT4)_3_

To a solution of TRAP(alkyne)_3_ (1.75 mg, 2.3 µmol, 1.0 eq) [21] and sodium ascorbate (4.6 mg, 23 µmol, 10 eq) in water (50 µL), solutions of azido-NT (8.0 mg, 7.6 µmol, 3.3 eq) in MeOH (200 µL) and Cu(OAc)_2_ (0.56 mg, 2.8 µmol, 1.2 eq) in water (50 µL) were added and the greenish mixture stirred for 1 h. Then, a solution of 1,4,7-triazacyclononane-1,4,7-triacetic acid (NOTA, 8.4 mg, 27.6 µmol, 12 eq) in dilute aqueous HCl (4 mL, 1 µM, pH 3.0) was added and left to react for 2 days at ambient temperature. This mixture was subjected to preparative HPLC purification (column: 250 × 10 mm C18, gradient: 21%–44% MeCN in water, both containing 0.1% trifluoroacetic acid, within 20 min). Concentration in vacuo and lyophilisation yielded the trimer as a colorless solid (3.68 mg, 0.97 µmol, 42%). Analytical HPLC: Nucleosil C18 5 µm, 1 mL/min, linear gradient 20%–70% CH_3_CN in H_2_O + 0.1% TFA in 20 min, 220 nm, purity: >95% (*t*_R_: 13.6 min). ESI-MS: [M + H]^+^; calcd for∙C_159_H_268_N_42_O_36_P_3_: 3437.0, found: 1719.2 [M + 2H]^2+^, 1146.3 [M + ^3^H]^3+^.

### 4.4. Synthesis of Ga-TRAP(NT4)_3_

Equimolar amounts of TRAP(NT4)_3_ and Ga(NO_3_)_3_ (in the form of 2 mM aq. solutions) were mixed at ambient temperature, whereupon the complex was immediately formed. The solution was lyophilized and the colorless solid used for in vitro experiments without further purification. ESI-MS: [M + H]^+^; calcd for C_159_H_265_N_42_O_36_P_3_Ga: 3501.9, found: 1752.0 [M + 2H]^2+^, 1168.5 [M + ^3^H]^3+^.

### 4.5. Radiosynthesis of [^68^Ga]Ga-TRAP(NT4)_3_

[^68^Ga]GaCl_3_ (110–140 MBq) was eluted from a ^68^Ge/^68^Ga generator (ITG Isotope Technologies Garching, Garching, Germany) with 50 mM HCl onto a cation exchanger cartridge (Chromafix PS H^+^, Macherey-Nagel, Düren, Germany), from which it was eluted with 1 mL NaCl (5 M). The solution was adjusted to a pH of 2.5–3 with HEPES buffer (2.5 M, 200 μL) and 1 nmol TRAP(NT4)_3_ was added. After 5 min at 98 °C the radiochemical yield (RCY) was >95% as determined by radio-HPLC (Chromolith RP-18e, 10 × 4.6 mm, 10%–100% CH_3_CN in H_2_O (0.1% TFA) in a linear gradient over 5 min, 4 mL/min, *t*_R_ = 1.77 min). The product was isolated by SPE (Sep-Pak C18 Plus Light Cartridge, Waters, Eschborn, Germany) and formulated with 0.9% saline. The apparent molar radioactivities at EOS were between 80 and 120 MBq/nmol.

### 4.6. Receptor Binding Assays

Receptor binding data were performed as described previously [31]. In detail, NTS1 binding was determined using homogenates of membranes from CHO cells stably expressing human NTS1 at a final concentration of 2 µg membrane protein/well and the radioligand [^3^H]NT(8–13) (specific activity 136 Ci/mmol; custom synthesis of [leucine-^3^H]NT(8–13) by GE Healthcare, Freiburg, Germany) at a concentration of 0.50 nM. Specific binding of the radioligand was determined at a K_D_ value of 0.37 nM and a B_max_ of 2500 fmol/mg protein. NTS2 binding was conducted using homogenates of membranes from HEK 293 cells transiently transfected with pcDNA3.1 carrying the human NTS2 gene (Missouri S&T cDNA Resource Center (UMR), Rolla, MO, USA) using the Mirus TransIT-293 transfection reagent (MoBiTec, Göttingen, Germany) according to the manufacturer’s protocol. Membranes were incubated at a final concentration of 6–20 µg/well and with 0.50 nM of [^3^H]NT(8–13) at K_D_ values of 0.87–1.3 nM and B_max_ values of 450–1500 fmol/mg protein. Nonspecific binding for both receptor subtypes was determined in the presence of 10 µM NT(8–13) and the protein concentration was determined by the method of Lowry using bovine serum albumin as standard [42].

Data analysis of the competition binding curves from the radioligand displacement experiments was done by non-linear regression analysis when applying the algorithms of the PRISM 6.0 software package (GraphPad Software, San Diego, CA, USA). EC_50_ values were derived from each dose-response curve and were subsequently transformed into the corresponding K_i_ values by applying the equation of Cheng and Prusoff [43]. Mean K_i_ values and the corresponding S.E.M. values (standard error of the mean) were determined by analyzing four to eight individual experiments.

### 4.7. Cell Culture

The human NTS1 expressing colon cancer cell line HT29 (ECACC no. 91072201) was grown in culture medium (McCoy’s 5a medium containing glutamine (2 mM) supplemented with fetal bovine serum (FBS, 10%)) at 37 °C in a humidified atmosphere of 5% CO_2_. Cells were routinely subcultured every 3−4 days. Routine tests of the HT29 cells for contamination with mycoplasma were always negative.

### 4.8. Internalization and Efflux

Internalization and efflux experiments were conducted using HT29 cells in 24-multiwell plates and 0.3 MBq of radiotracer as described before [27]. The experiments were performed in quadruplicate and were repeated at least twice.

### 4.9. Animal Model

All animal experiments were performed in compliance with the protocols approved by the local Animal Protection Authorities (Regierung Mittelfranken, Germany, no. 54-2532.1-15/08). Female athymic NMRI nude mice (nu/nu) were obtained from Harlan Winkelmann GmbH (Borchen, Germany) at four weeks of age and were kept under standard conditions (12 h light/dark) with food and water available ad libitum for at least five weeks. HT29 tumors were generated as described before [18].

### 4.10. Biodistribution Studies

HT29 xenografted nude mice were injected with [^68^Ga]Ga-TRAP(NT4)_3_ into a tail vein (2.5–7.5 MBq/mouse). The animals were killed by cervical dislocation 60 or 90 min p.i. The tumors, other tissues (lung, liver, kidneys, heart, spleen, brain, muscle, femur, and intestine) and blood were removed and weighed. Radioactivity of the samples was measured using a γ-counter and expressed as the percentage of injected dose per gram of tissue (%ID/g), from which tumor-to-blood ratios were calculated. Blocking experiments were carried out by co-injecting randomly chosen mice with TRAP(NT4)_3_ (20 nmol/mouse) or NT4 (100 nmol/mouse) together with the radiotracer. These mice were killed by cervical dislocation at 60 min p.i., and organs and tissue were removed, weighed, and counted as described above.

### 4.11. Small Animal PET Imaging

PET scans and image analysis were performed using a small-animal PET scanner (Inveon, Siemens Medical Solutions, Erlangen, Germany). About 1.5–4 MBq of [^68^Ga]Ga-TRAP(NT4)_3_ with varying amounts of 0.02–10 nmol TRAP(NT4)_3_ were intravenously injected into the mice under isoflurane anesthesia (4%). Animals were subjected to a 60 min dynamic scan starting with tracer injection. After iterative maximum a posteriori image reconstruction of the decay- and attenuation-corrected images, regions of interest (ROIs) were drawn over the tumor. The radioactivity concentration within the tumor region was obtained from the mean value within the multiple ROIs and then converted to SUV relative to the injected dose and the individual body weight.

## 5. Conclusions

We successfully synthesized the trimeric PET ligand [^68^Ga]Ga-TRAP(NT4)_3_ using the novel chelator TRAP and evaluated the radioligand in vitro and in vivo. [^68^Ga]Ga-TRAP(NT4)_3_ revealed two-fold tumor uptake compared to monomeric analogs, however, the biodistribution of the trimer [^68^Ga]Ga-TRAP(NT4)_3_ showed limitations due to high uptake and slow washout in excretion organs. Therefore, the multimerization approach in the case of [^68^Ga]Ga-TRAP(NT4)_3_ for neurotensin receptor imaging did not result in a PET ligand with significantly improved in vivo properties compared to a corresponding monomer radioligand.

In order to assess the potential of multimerization with utmost independence of scaffold-related effects, TRAP-based trimers and structurally-related monomeric chelator conjugates of neurotensin ligands and other targeting vectors (peptids and peptoids targeting integrins α_v_β_3_, α_5_β_1_, and α_v_β_6_, the PSMA-ligand DUPA, and nitroimidazoles targeting hypoxia) were compared in terms of in vitro and in vivo properties. We found that:

1. trimerization invariably effected enhancement of target affinities and tumor uptakes;
2. trimerization had a highly variable influence on tumor-to-organ ratios, ranging from substantial improvement to strong deterioration; and
3. there is no correlation of in vitro data (affinity, log *D*) with in vivo performance.

We conclude that although multimers can be expected to exhibit higher target affinities, these data are not suitable for prediction of in vivo behavior, and multimeric structures must always be individually tested in vivo. Notwithstanding this, their consistently higher tumor uptake renders them potentially superior to monomers, particularly for applications where high tumor accumulation is crucial, such as for therapeutics.

## Figures and Tables

**Table 1:** In vitro binding affinities of Ga-TRAP(NT4)_3_ to the human NTS1 and NTS2 ^1^.

Compound	K_i_ (NTS1, nM)	K_i_ (NTS2, nM)
NT(8–13)	0.29 ± 0.03 ^2^	1.40 ± 0.11 ^2,3^
Pra-NLys-Lys-Pro-Tyr-Tle-Leu-OH (NT4)	4.6 ± 0.64	51 ± 10
TRAP(NT4)_3_	0.47 ± 0.05	0.26 ± 0.05
Ga-TRAP(NT4)_3_	0.12 ± 0.03	0.21 ± 0.05

^1^ Data are expressed as mean values ± SEM (standard error of the mean) from four to eight independent experiments each determined in triplicate; ^2^ Values from [18] for comparison; ^3^ K_D_ value ± SEM determined with the radioligand [^3^H]NT(8–13) in saturation binding experiments each performed in quadruplicates.

**Table 2:** Biodistribution data of [^68^Ga]Ga-TRAP(NT4)_3_ expressed as percent injected dose per gram tissue (%ID/g) ± standard deviation of 2–3 HT29 tumor-bearing nude mice for each time point.

Organ	60 min	90 min	60 min Blocking ^1^	60 min Blocking ^2^
blood	0.13 ± 0.02	0.04 ± 0.02	0.07 ± 0.01	0.40 ± 0.01
lung	2.76 ± 0.53	2.37 ± 0.21	1.61 ± 0.42	6.06 ± 1.19
liver	11.18 ± 0.30	11.71 ± 0.48	8.30 ± 0.87	11.21 ± 2.27
kidney	94.55 ± 10.84	102.37 ± 29.88	96.26 ± 8.83	99.91 ± 15.52
heart	0.13 ± 0.02	0.35 ± 0.37	0.15 ± 0.11	0.45 ± 0.13
spleen	3.72 ± 0.09	3.45 ± 0.38	2.20 ± 0.39	3.75 ± 0.81
brain	0.04 ± 0.01	0.02 ± 0.01	0.06 ± 0.07	0.07 ± 0.02
muscle	0.09 ± 0.03	0.11 ± 0.08	0.10 ± 0.05	0.16 ± 0.03
femur	1.51 ± 0.12	1.17 ± 0.21	0.74 ± 0.14	1.72 ± 0.12
HT29 tumor	1.74 ± 0.21	1.44 ± 0.13	1.10 ± 0.20	0.60 ± 0.13
intestine	1.50 ± 0.42	1.31 ± 0.20	0.49 ± 0.08	0.49 ± 0.07

^1^ Co-injection of [^68^Ga]Ga-TRAP(NT4)_3_ and TRAP(NT4)_3_ (20 nmol per mouse); ^2^ Co-injection of [^68^Ga]Ga-TRAP(NT4)_3_ and NT4 (100 nmol per mouse).

**Table 3:** Comparison of affinities, octanol/PBS distribution coefficients, and in vivo biodistribution data for selected TRAP-based trimers and monomeric chelator conjugates of the respective same targeting vectors, selected according to structural equivalence and availability of data for the same tumor models. Data were measured for the respective ^68^Ga or ^nat^Ga complexes, respectively, except those marked with an asterisk (*). Errors (± standard deviation) are given for data first reported herein, but are omitted for all other figures taken from the literature.

Ga-Monomer		NODAGA-PEG6-NT4		NOPO-RGD	NODAGA-FR366	TRAP(AvB6)_1_ (Avebehexin)	DOTAGA-DUPA	TRAP(NIm)_1_
Ga-Trimer		TRAP(NT4)_3_		TRAP(RGD)_3_ (Avebetrin)	TRAP(FR366)_3_ (Aquibeprin)	TRAP(AvB6)_3_	TRAP(DUPA)_3_	TRAP(NIm)_3_
**Targeting vector**		NTS1-selective peptoid (Pra-NLys-Lys-Pro-Tyr-Tle-Leu-OH (NT4))		c(RGDfK)	α5β1-integrin- selective peptoid FR366	c(FRGDLAF-p[*N*Me]K)	DUPA-Pep (EuK-C_8_-Phe-Phe)	Nitro -imidazole
**Target**		NTS1	NTS2	αvβ3 integrin	α5β1 integrin	αvβ6 integrin	PSMA	hypoxia
**Affinity** IC_50_ or ^#^Ki (nM)	Monomer	20 ^#^	87 ^#^	1.1	1.3 *	0.26	36	n/a
	Trimer	0.12 ^#^	0.20 ^#^	0.22	0.083	0.023	2	n/a
	**factor**	**166**	**435**	**5**	**16**	**11**	**18**	n/a
**log *D*** pH 7.4	Monomer	−4.1		−4.6	−3.9	−3.7	−3.6 ± 0.2	n/a
	Trimer	−3.7 ± 0.1		−3.9	−4.2	−1.7	−2.9 ± 0.1	−3.3
***Xenograft/Time p.i.***		*HT29/60 min*		*M21/120 min*	*M21/90 min*	*H2009/90 min*	*LNCaP/60 min*	*CT26/60 min*
**Tumor** %ID/g	Monomer	1.55		1.4	0.64	0.65	2.0 ± 0.2	0.33
	Trimer	1.74		4.6	2.4	0.92 ± 0.08	6.7 ± 1.9	0.47
**Blood** %ID/g	Monomer	0.05		0.04	0.07	0.17	2.5 ± 0.14	0.41
	Trimer	0.13		0.16	0.24	1.9 ± 0.15	0.41 ± 0.18	0.52
**Muscle** %ID/g	Monomer	0.10		0.22	0.04	0.06	1.2 ± 0.13	0.17
	Trimer	0.09		0.66	0.12	0.71 ± 0.10	0.12 ± 0.03	0.20
**Liver** %ID/g	Monomer	0.3		1.6	0.32	0.36	2.0 ± 0.17	0.24
	Trimer	11		3.9	0.48	3.7 ± 0.14	0.51 ± 0.10	0.34
**Kidney** %ID/g	Monomer	45		1.9	1.2	4.3	29 ± 7	2.06
	Trimer	95		8.6	8.0	17.7 ± 6.5	138 ± 11	2.72
**Remarks**				All data for high molar activities (1–2 GBq/µmol)		Trimer data estimated from PET ROI analysis	Monomer data estimated from PET ROI analysis	
References		[18], this work		[22,37]	[38,39,40]	[34]	[21]	[41]

## References

[1] Dupouy S., Mourra N., Gompel A., Alifano M., Forgez P. (2011). The potential use of the neurotensin high affinity receptor 1 as a biomarker for cancer progression and as a component of personalized medicine in selective cancers. Biochimie.

[2] Reubi J., Waser B., Friess H., Büchler M., Laissue J. (1998). Neurotensin receptors: A new marker for human ductal pancreatic adenocarcinoma. Gut.

[3] Alifano M., Souazé F., Dupouy S., Camilleri-Broët S., Younes M., Ahmed-Zaïd S.-M., Takahashi T., Cancellieri A., Damiani S., Boaron M. (2010). Neurotensin receptor 1 determines the outcome of non–small cell lung cancer. Clin. Cancer Res..

[4] Souazé F., Dupouy S., Viardot-Foucault V., Bruyneel E., Attoub S., Gespach C., Gompel A., Forgez P. (2006). Expression of neurotensin and NT1 receptor in human breast cancer: A potential role in tumor progression. Cancer Res..

[5] Swift S.L., Burns J.E., Maitland N.J. (2010). Altered expression of neurotensin receptors is associated with the differentiation state of prostate cancer. Cancer Res..

[6] Morgat C., Mishra A.K., Varshney R., Allard M., Fernandez P., Hindié E. (2014). Targeting neuropeptide receptors for cancer imaging and therapy: Perspectives with bombesin, neurotensin, and neuropeptide-Y receptors. J. Nucl. Med..

[7] Wu Z., Martinez-Fong D., Trédaniel J., Forgez P. (2013). Neurotensin and its high affinity receptor 1 as a potential pharmacological target in cancer therapy. Front. Endocrinol..

[8] Aronin N., Carraway R.E., Ferris C.F., Hammer R.A., Leeman S.E. (1982). The stability and metabolism of intravenously administered neurotensin in the rat. Peptides.

[9] Lee Y.C., Uttenthal L.O., Smith H.A., Bloom S.R. (1986). In vitro degradation of neurotensin in human plasma. Peptides.

[10] Orwig K.S., Lassetter M.R., Hadden M.K., Dix T.A. (2009). Comparison of N-Terminal Modifications on Neurotensin(8–13) Analogues Correlates Peptide Stability but Not Binding Affinity with in Vivo Efficacy. J. Med. Chem..

[11] Sparr C., Purkayastha N., Yoshinari T., Seebach D., Maschauer S., Prante O., Hübner H., Gmeiner P., Kolesinska B., Cescato R. (2013). Syntheses, Receptor Bindings, in vitro and in vivo Stabilities and Biodistributions of DOTA-Neurotensin(8–13) Derivatives Containing β-Amino Acid Residues—A Lesson about the Importance of Animal Experiments. Chem. Biodivers..

[12] Mascarin A., Valverde I.E., Mindt T.L. (2016). Structure-Activity Relationship Studies of Amino Acid Substitutions in Radiolabeled Neurotensin Conjugates. ChemMedChem.

[13] Bruehlmeier M., Garayoa E.G., Blanc A., Holzer B., Gergely S., Tourwe D., Schubiger P.A., Blauenstein P. (2002). Stabilization of neurotensin analogues: Effect on peptide catabolism, biodistribution and tumor binding. Nucl. Med. Biol..

[14] Charron C., Hickey J., Nsiama T., Cruickshank D., Turnbull W., Luyt L. (2016). Molecular imaging probes derived from natural peptides. Nat. Prod. Rep..

[15] Rahmim A., Zaidi H. (2008). PET versus SPECT: Strengths, limitations and challenges. Nucl. Med. Commun..

[16] Maschauer S., Einsiedel J., Hocke C., Hübner H., Kuwert T., Gmeiner P., Prante O. (2010). Synthesis of a ^68^Ga-labeled peptoid-Peptide hybrid for imaging of neurotensin receptor expression in vivo. ACS Med. Chem. Lett..

[17] Maschauer S., Einsiedel J., Haubner R., Hocke C., Ocker M., Hübner H., Kuwert T., Gmeiner P., Prante O. (2010). Labeling and glycosylation of peptides using click chemistry: A general approach to ^18^F-glycopeptides as effective imaging probes for positron emission tomography. Angew. Chem. Int. Ed. Engl..

[18] Maschauer S., Einsiedel J., Hübner H., Gmeiner P., Prante O. (2016). ^18^F- and ^68^Ga-Labeled Neurotensin Peptides for PET Imaging of Neurotensin Receptor 1. J. Med. Chem..

[19] Röhrich A., Bergmann R., Kretzschmann A., Noll S., Steinbach J., Pietzsch J., Stephan H. (2011). A novel tetrabranched neurotensin(8–13) cyclam derivative: Synthesis, ^64^Cu-labeling and biological evaluation. J. Inorg. Biochem..

[20] Hultsch C., Berndt M., Bergmann R., Wuest F. (2007). Radiolabeling of multimeric neurotensin(8–13) analogs with the short-lived positron emitter fluorine-18. Appl. Radiat. Isot..

[21] Baranyai Z., Reich D., Vagner A., Weineisen M., Toth I., Wester H.J., Notni J. (2015). A shortcut to high-affinity Ga-68 and Cu-64 radiopharmaceuticals: One-pot click chemistry trimerisation on the TRAP platform. Dalton Trans..

[22] Notni J., Pohle K., Wester H.J. (2013). Be spoilt for choice with radiolabelled RGD peptides: Preclinical evaluation of ^68^Ga-TRAP(RGD)_3_. Nucl. Med. Biol..

[23] Notni J., Pohle K., Wester H.J. (2012). Comparative gallium-68 labeling of TRAP-, NOTA-, and DOTA-peptides: Practical consequences for the future of gallium-68-PET. EJNMMI Res..

[24] Simecek J., Schulz M., Notni J., Plutnar J., Kubicek V., Havlickova J., Hermann P. (2012). Complexation of metal ions with TRAP (1,4,7-triazacyclononane phosphinic acid) ligands and 1,4,7-triazacyclononane-1,4,7-triacetic acid: Phosphinate-containing ligands as unique chelators for trivalent gallium. Inorg. Chem..

[25] Notni J., Simecek J., Hermann P., Wester H.J. (2011). TRAP, a Powerful and Versatile Framework for Gallium-68 Radiopharmaceuticals. Chem. Eur. J..

[26] Einsiedel J., Hübner H., Hervet M., Harterich S., Koschatzky S., Gmeiner P. (2008). Peptide backbone modifications on the C-terminal hexapeptide of neurotensin. Bioorg. Med. Chem. Lett..

[27] Maschauer S., Ruckdeschel T., Tripal P., Haubner R., Einsiedel J., Hübner H., Gmeiner P., Kuwert T., Prante O. (2014). In vivo monitoring of the antiangiogenic effect of neurotensin receptor-mediated radiotherapy by small-animal positron emission tomography: A pilot study. Pharmaceuticals.

[28] Notni J., Hermann P., Havlickova J., Kotek J., Kubicek V., Plutnar J., Loktionova N., Riss P.J., Rösch F., Lukes I. (2010). A triazacyclononane-based bifunctional phosphinate ligand for the preparation of multimeric ^68^Ga tracers for positron emission tomography. Chem. Eur. J..

[29] Notni J., Simecek J., Wester H.J. (2014). Phosphinic acid functionalized polyazacycloalkane chelators for radiodiagnostics and radiotherapeutics: Unique characteristics and applications. ChemMedChem.

[30] Notni J., Wester H.J. (2016). A Practical Guide on the Synthesis of Metal Chelates for Molecular Imaging and Therapy by Means of Click Chemistry. Chem. Eur. J..

[31] Lang C., Maschauer S., Hübner H., Gmeiner P., Prante O. (2013). Synthesis and evaluation of a ^18^F-labeled diarylpyrazole glycoconjugate for the imaging of NTS1-positive tumors. J. Med. Chem..

[32] Jia Y., Zhang W., Fan W., Brusnahan S., Garrison J. (2016). Investigation of the Biological Impact of Charge Distribution on a NTR1-Targeted Peptide. Bioconj. Chem..

[33] Hübner H., Schellhorn T., Gienger M., Schaab C., Kaindl J., Leeb L., Clark T., Möller D., Gmeiner P. (2016). Structure-guided development of heterodimer-selective GPCR ligands. Nat. Commun..

[34] Notni J., Reich D., Maltsev O.V., Kapp T.G., Steiger K., Hoffmann F., Esposito I., Weichert W., Kessler H., Wester H.-J. (2017). In Vivo PET imaging of the “cancer integrin” αvβ6 using gallium-68 labelled cyclic RGD nonapeptides. J. Nucl. Med..

[35] Weineisen M., Simecek J., Schottelius M., Schwaiger M., Wester H.J. (2014). Synthesis and preclinical evaluation of DOTAGA-conjugated PSMA ligands for functional imaging and endoradiotherapy of prostate cancer. EJNMMI Res..

[36] Bacher L., Fischer G., Litau S., Schirrmacher R., Wängler B., Baller M., Wängler C. (2015). Improving the stability of peptidic radiotracers by the introduction of artificial scaffolds: Which structure element is most useful?. J. Label. Compd Radiopharm..

[37] Simecek J., Notni J., Kapp T.G., Kessler H., Wester H.J. (2014). Benefits of NOPO as chelator in gallium-68 peptides, exemplified by preclinical characterization of ^68^Ga-NOPO-c(RGDfK). Mol. Pharm..

[38] Notni J., Steiger K., Hoffmann F., Reich D., Kapp T.G., Rechenmacher F., Neubauer S., Kessler H., Wester H.J. (2016). Complementary, Selective PET Imaging of Integrin Subtypes α5β1 and αvβ3 Using ^68^Ga-Aquibeprin and ^68^Ga-Avebetrin. J. Nucl. Med..

[39] D‘Alessandria C., Pohle K., Rechenmacher F., Neubauer S., Notni J., Wester H.J., Schwaiger M., Kessler H., Beer A.J. (2016). In vivo biokinetic and metabolic characterization of the ^68^Ga-labelled α5β1-selective peptidomimetic FR366. Eur. J. Nucl. Med. Mol. Imaging.

[40] Notni J., Steiger K., Hoffmann F., Reich D., Schwaiger M., Kessler H., Wester H.J. (2016). Variation of Specific Activities of ^68^Ga-Aquibeprin and ^68^Ga-Avebetrin Enables Selective PET Imaging of Different Expression Levels of Integrins α5β1 and αvβ3. J. Nucl. Med..

[41] Seelam S.R., Lee J.Y., Lee Y.S., Hong M.K., Kim Y.J., Banka V.K., Lee D.S., Chung J.K., Jeong J.M. (2015). Development of ^68^Ga-labeled multivalent nitroimidazole derivatives for hypoxia imaging. Bioorg. Med. Chem..

[42] Lowry O.H., Rosebrough N.J., Farr A.L., Randall R.J. (1951). Protein measurement with the Folin phenol reagent. J. Biol. Chem..

[43] Cheng Y., Prusoff W.H. (1973). Relationship between the inhibition constant (K1) and the concentration of inhibitor which causes 50 per cent inhibition (I50) of an enzymatic reaction. Biochem. Pharmacol..

